# Hippocampal Functioning and Verbal Associative Memory in Adolescents with Congenital Hypothyroidism

**DOI:** 10.3389/fendo.2015.00163

**Published:** 2015-10-19

**Authors:** Sarah M. Wheeler, Victoria C. McLelland, Erin Sheard, Mary Pat McAndrews, Joanne F. Rovet

**Affiliations:** ^1^Neurosciences and Mental Health Research Program, The Hospital for Sick Children, Toronto, ON, Canada; ^2^Department of Psychology, University of Toronto, Toronto, ON, Canada; ^3^Krembil Neuroscience Centre, Toronto Western Hospital, Toronto, ON, Canada; ^4^Department of Pediatrics, University of Toronto, Toronto, ON, Canada

**Keywords:** congenital hypothyroidism, thyroid hormone, verbal associative memory, fMRI, hippocampus

## Abstract

Thyroid hormone (TH) is essential for normal development of the hippocampus, which is critical for memory and particularly for learning and recalling associations between visual and verbal stimuli. Adolescents with congenital hypothyroidism (CH), who lack TH in late gestation and early life, demonstrate weak verbal recall abilities, reduced hippocampal volumes, and abnormal hippocampal functioning for visually associated material. However, it is not known if their hippocampus functions abnormally when remembering verbal associations. Our objective was to assess hippocampal functioning in CH using functional magnetic resonance imaging (fMRI). Fourteen adolescents with CH and 14 typically developing controls (TDC) were studied. Participants studied pairs of words and then, during fMRI acquisition, made two types of recognition decisions: in one they judged whether the *pairs* were the same as when seen originally and in the other, whether *individual words* were seen before regardless of pairing. Hippocampal activation was greater for pairs than items in both groups, but this difference was only significant in TDC. When we directly compared the groups, the right anterior hippocampus was the primary region in which the TDC and CH groups differed for this pair memory effect. Results signify that adolescents with CH show abnormal hippocampal functioning during verbal memory processing.

## Introduction

The hippocampus, which is essential for learning and memory (1), needs thyroid hormone (TH) for normal development (2). Rodents with early TH deprivation show reduced size (3) and atypical hippocampal structure and functioning (4–10) and have learning and memory deficits (11, 12). Children with congenital hypothyroidism (CH) who experience neonatal TH deficiency, albeit generally of shorter duration than in many animal models, exhibit mild IQ reductions (13) and cognitive deficits (14) on memory tasks involving the hippocampus (15, 16).

We previously described smaller hippocampal volumes in CH adolescents relative to controls, particularly on the left side, and their hippocampal volumes were correlated with their verbal memory performance (17). Using functional magnetic resonance imaging (fMRI), we observed CH adolescents relative to controls showed increased bilateral hippocampal engagement when recognizing paired objects and increased activation in the left hippocampus for objects’ locations (18). Given links between the left hippocampus and verbal associative processing (19) and our observation of greater volume reductions on the left than right hippocampus in CH adolescents (17), we currently investigated if they also show abnormal hippocampal functioning when remembering verbal associations. This was accomplished using a task shown in adults to preferentially engage the left hippocampus when recognizing word pairs relative to recognizing individual words (20). Based on our previous fMRI findings, we hypothesized that CH would show increased hippocampal activation for word pairs relative to controls and that this effect would reflect initial disease severity.

## Materials and Methods

### Participants

Seventeen adolescents with CH and 17 age-matched typically developing controls (TDC) were recruited (see Table 1 for demographic and biomedical information). None had MRI contraindications (braces, metal implants, or metal filing exposure).

**Table 1:** **Demographic and biomedical information for CH and TDC groups**.

	CH (*n* **=** 14)	TDC (*n* **=** 14)	*p*
Age at testing [mean ± SD; (range)]	13.4 ± 1.0 (11.5–14.7)	12.9 ± 1.5 (11.2–15.6)	0.25
Sex (% male)	57.1	57.1	0.71
Handedness (% left handed)	7.1	14.3	0.54
WASI Full Scale IQ (mean ± SD)	105.1 ± 8.0	109.3 ± 9.3	0.38
Age at diagnosis [median (range)]	14 (7 days to 2 years)		
Levels at diagnosis			
Median TSH (range)	433 mU/L (18–1072)		
Free T4 (mean ± SD)	8.52 ± 4.5 pmol/L		
Total T4 (mean ± SD)	50.4 ± 39.2 nmol/L		

The CH group consisted of 5 cases from a birth cohort, 2 past participants (21), and 10 new recruits through the SickKids Endocrine Clinic. One child was eliminated for poor compliance, another for MRI data corruption, and a third for pseudohypoparathyroidism diagnosed from the MRI, resulting in a final sample size of eight males and six females (mean age = 13.4 ± 1.0 years; 13 right-handed). Twelve children were detected via the Ontario newborn screening program, which uses a thyroid stimulating hormone (TSH) test, while the remaining two included a boy diagnosed clinically in Ontario at 62 days because the courier company lost his newborn sample and a boy born abroad, where screening was not performed, who was followed since infancy at SickKids. The median diagnostic age was 14 days. Nine screened cases received treatment within 2 weeks of birth and three with borderline TSH levels and normal-range T4 values were treated once their T4 levels declined at about 1 month of age. Technetium scans performed at the initial clinic visit showed two with athyreosis, eight with an ectopic gland, two with dyshormonogenesis, and two with unknown etiologies because parents refused the scan or child was born abroad. The median start dose of L-T4 was 9.96 mg/kg/day (range = 8.5–10.8).

The TDC group consisted of nine from the same birth cohort, seven past participants (15), and one new child ascertained via a hospital poster. Exclusionary criteria were prenatal teratogen exposure, preterm birth, head injury, chronic medical or neurological conditions including hypothyroidism, and learning disability or psychiatric disorder. One child was eliminated for a significant neuroradiological abnormality and two for excessive movement during scanning for a final sample size of eight males and four females (mean age = 12.9 ± 1.5 years; 12 right-handed). Screening TSH values known for cases from the birth cohort were all normal. In all TDC, family history was negative for thyroid disease.

### Procedures

This study was part of a larger project in which participants were tested on 2 days several weeks apart. On day one, they received a battery of clinical tests and on day 2, a 1-h morning visuospatial fMRI session (18) and afternoon session with the current verbal memory paradigm. Participants received a CD of their brain scan, certificate for school volunteer hours, and movie pass. Parents were compensated for transportation costs. A neuroradiologist masked to group status examined MRI images and the child’s pediatrician was informed of abnormalities. Procedures were approved by the ethics boards of SickKids and University of Toronto.

### Tests and Measures

#### Intelligence

The Wechsler Abbreviated Scale of Intelligence (WASI) two-subtest version consisting of Vocabulary and Matrix Reasoning subtests was administered (22).

#### Verbal Paired Associates Paradigm

An adult word-pairs paradigm (20) was modified to be age-appropriate by presenting tasks as “games,” using more juvenile stimuli, reducing the number of word pairs, and lengthening stimulus presentation time. Words were selected from the MRC Psycholinguistic Database (23) to have (i) a length of four to six letters and one or two syllables, (ii) a score greater than or equal to the mean in concreteness and imageability, (iii) Thorndike-Lorge written frequency of AA (appearing at least 100 times per million words written), and (iv) acquisition before age four. Two independent raters examined words for age-appropriateness and any deemed inappropriate were removed. Of the 441 words identified, 361 were randomly selected. For each participant, words were randomly paired from the list and unique sets were used in pretraining and fMRI sessions.

All participants received pre-training outside the scanner to a criterion of 10 sequential correct responses per trial type. They were shown sample cards to illustrate the “games” followed by a computer pre-training task with encoding and retrieval phases. During encoding, they saw word pairs and had to silently generate detailed sentences combining each pair of words in order shown, a device shown to effectively promote associative binding and subsequent hippocampal engagement in later recollection (24, 25). During retrieval, participants received two tasks, Pair Recognition and Item Recognition, each signaled by a distinctive cue. Both tasks presented word pairs but differed as to question asked: Pair Recognition required them to respond YES if words were *paired* during encoding and NO otherwise; Item Recognition required a YES response if both words were *studied before regardless of pairing* and NO if at least one word was new. The control task, which matched the others in cueing, layout of stimuli, and responses but lacked a memory component, required participants to use the YES/NO keys to signal the side where an “@” symbol appeared; control trials were randomly interspersed between other trials.

In the scanner, stimuli were presented using E-Prime software via MR-compatible goggles. Participants responded using a two-button MR-compatible response pad with left/right placement of YES and NO keys counterbalanced across subjects. Four runs each had an encoding and retrieval phase. During encoding, 14 word pairs were successively presented for 7 s each, to allow for silent sentence generation. During retrieval, eight 44-s blocks alternating between Pair Recognition and Item Recognition tasks were presented sequentially. Each task was preceded by a 5-s slide showing the task logo, instructions, and response-button reminder and followed by 12 trials; retrieval trials lasted 4 s each, while control trials were of varying length (2, 2.5, or 3 s) to introduce jitter. Task order was counterbalanced across participants.

On Pair-Recognition trials, participants saw: (i) intact word pairs as in encoding, (ii) rearranged pairs with the words shown in encoding but in different combinations, and (iii) new pairs not seen in encoding. In this task, the YES button was accurate only for correct intact pairs. On Item-Recognition trials, participants saw: (i) two words from the encoding list but in a different combination, (ii) pairs with one old and one new word, or (iii) pairs with two new words. Here, the YES button was accurate if *both* items regardless of pairing were seen before. In each block, three trials required a YES response (i.e., intact pairs or rearranged items) and six a NO response while there were three control trials. Trial order was pseudo-randomized within blocks, with no more than two trials of one type presented consecutively and a control trial always appearing last.

### Data Acquisition and Analysis

fMRI data were acquired on a 1.5 T Signa GE scanner at SickKids using echo-planar imaging (TR = 2 s, 25 slices, field of view = 240 mm, matrix size = 64 × 64, voxel size = 3.75 mm × 3.75 mm × 5 mm) and an 8-channel head coil. Slices had a coronal-oblique orientation, perpendicular to the hippocampus’s long axis. Two hundred functional volumes were collected in each of the four retrieval runs. To allow for signal equilibrium, the first three volumes were discarded. E-Prime software simultaneously recorded accuracy and reaction time (RT) for each trial.

Pre-processing and fMRI analyses were conducted in SPM5 (Statistical Parametric Mapping 5; Wellcome Trust Centre for Neuroimaging). Runs with movement exceeding ±1 mm and/or ±1° rotation from baseline had relevant scans removed. Any run showing excessive motion throughout was discarded. Pre-processing included: (i) realigning for motion correction, (ii) slice-time correction to the middle slice, (iii) normalizing brains to the Montreal Neurological Institute EPI template, resampling at a voxel size of 4 mm× 4 mm × 4 mm, and (iv) smoothing with a Gaussian kernel of 8 mm full-width half maximum to improve signal-to-noise ratio. Each stimulus event was modeled by SPM5’s canonical hemodynamic response function beginning at stimulus onset.

Only correct trials were analyzed, since cognitive processes underlying brain activation during incorrect trials are unknown. Brain-activation differences between conditions were assessed at the first level using a planned contrast, as per the original adult study wherein greater hippocampal activation was found for recognizing *associations between words* than *individual words* (20). Accordingly, for each participant, we identified brain regions that were more active during all Pair-Recognition than all Item-Recognition trials. To identify hippocampal regions implicated for each group by the contrast, we performed second-level single-sample *t*-tests using age and mean accuracy as covariates. To examine for between-group differences, we used two-sample *t*-tests. Brains were masked to include only voxels within the hippocampus (our *a priori* region of interest) using the MAsks for Region of INterest Analysis software (MARINA; Bertram Walter Bender Institute of Neuroimaging). Lastly, to examine relationships between early disease indices and current hippocampal activation, we performed multiple regressions in SPM5 using age at diagnosis and diagnostic T4 and TSH values as regressors. For all analyses, the voxel-wise threshold was *p* < 0.005 and cluster size *k* > 5 voxels; we also note when results survive a more conservative threshold of *p* < 0.05 corrected for multiple comparisons using the family wise error rate.

For all figures, thresholded and hippocampal-masked activation maps from SPM were overlaid on a standard template (ch2better.nii) using MRIcron (26). Percentage signal change data were extracted for significant clusters using the REX toolbox in SPM5. MRI signal (β) values from each subject’s first-level model, which reflect the effect size for each condition, were averaged across all active voxels within a 2-mm sphere centered on each peak coordinate, and then were converted to percent signal change by dividing by the value of the run’s mean signal and multiplying it by 100. For each condition from each run, the percent signal change values were averaged to create one value per condition per subject. These were then used to compute the mean and SE for every condition.

Accuracy and RT behavioral data were analyzed in SPSS 22.0. Between-group comparisons were obtained using MANOVA with Bonferroni-corrected pairwise comparisons, while repeated measures analyses compared pair versus item tasks within groups. Correlations were performed between behavioral data and early CH variables.

## Results

Groups did not differ in age, handedness, sex, or IQ (Table 1). As also shown in Table 1, our CH sample had a very high median TSH value at diagnosis with low free T4 and total T4 levels.

A MANOVA with accuracy for each of the eight trial types as dependent variables revealed no significant main effect of group, but pairwise comparisons showed TDC were more accurate than CH on the intact pair (*p* = 0.02) and rearranged items trials (*p* = 0.01) and did not differ in accuracy for the remaining trial types (see Table 2). A similar MANOVA for RT again showed no main effect of group, but group differences on the rearranged items (*p* = 0.03), one new item (*p* = 0.02), and new item (*p* = 0.04) trials only. For RT, a significant group by task interaction (*p* < 0.05) reflected the tendency for TDC to respond faster on Pair than Item-Recognition trials (*p* = 0.02) and for CH to respond faster on Item than Pair trials, although this difference failed to reach significance. In TDC, accuracy and RT were negatively correlated for Pair (*r* = −0.55, *p* = 0.04) but not Item (*r* = −0.51, *p* = 0.06) trials, signifying better performance with shorter response times; these parameters were uncorrelated in CH (pair, *r* = −0.23, *ns*; Item, *r* = −0.29, *ns*). In CH, later age at treatment onset was negatively correlated with Pair-Recognition (*r* = −0.60, *p* = 0.02) and overall accuracy (*r* = −0.57, *p* = 0.03) but not Item-Recognition accuracy. No effects were observed for diagnostic T4 or TSH levels and task accuracy. All indices were unrelated to RT.

**Table 2:** **Mean **±** SD behavioral task results for CH and TDC groups**.

	CH (*n* **=** 14)	TDC (*n* **=** 14)	*F*	*p*	**η**^2^
**Accuracy (% correct)** **Pair recognition task**					
Intact pairs	58.5 ± 18.9	72.9 ± 11.8	5.91	0.02**	0.19
Rearranged pairs	69.2 ± 23.0	80.7 ± 10.6	2.87	0.10	0.10
New pairs	74.1 ± 26.5	88.4 ± 14.3	3.15	0.09	0.11
Pair control task	88.4 ± 10.7	89.6 ± 5.8	0.13	0.72	0.01
**Item-recognition task**					
Rearranged items	55.4 ± 10.1	67.6 ± 12.7	8.00	0.01**	0.24
One new item	68.2 ± 22.3	80.1 ± 15.3	2.72	0.11	0.10
New items	71.0 ± 28.1	88.4 ± 15.9	4.06	0.05	0.14
Item control task	88.5 ± 10.9	88.0 ± 4.1	0.04	0.85	0.001
**Reaction time (ms)** **Pair recognition task**					
Intact pairs	1560.63 ± 294.4	1683.0 ± 175.9	1.78	0.19	0.06
Rearranged pairs	1708.3 ± 282.8	1908.8 ± 334.6	2.93	0.10	0.10
New pairs	1489.8 ± 327.4	1645.3 ± 270.6	1.88	0.18	0.07
Pair control task	805.6 ± 108.4	832.3 ± 110.1	0.42	0.52	0.02
**Item-recognition task**					
Rearranged items	1570.1 ± 324.9	1842.2 ± 291.1	5.45	0.03**	0.17
One new item	1562.4 ± 381.9	1886.7 ± 290.6	6.39	0.02**	0.20
New items	1460.8 ± 328.9	1692.8 ± 238.2	4.57	0.04**	0.15
Item control task	801.2 ± 101.0	840.8 ± 123.9	0.86	0.36	0.03

**Significance.

**Figure 1:**
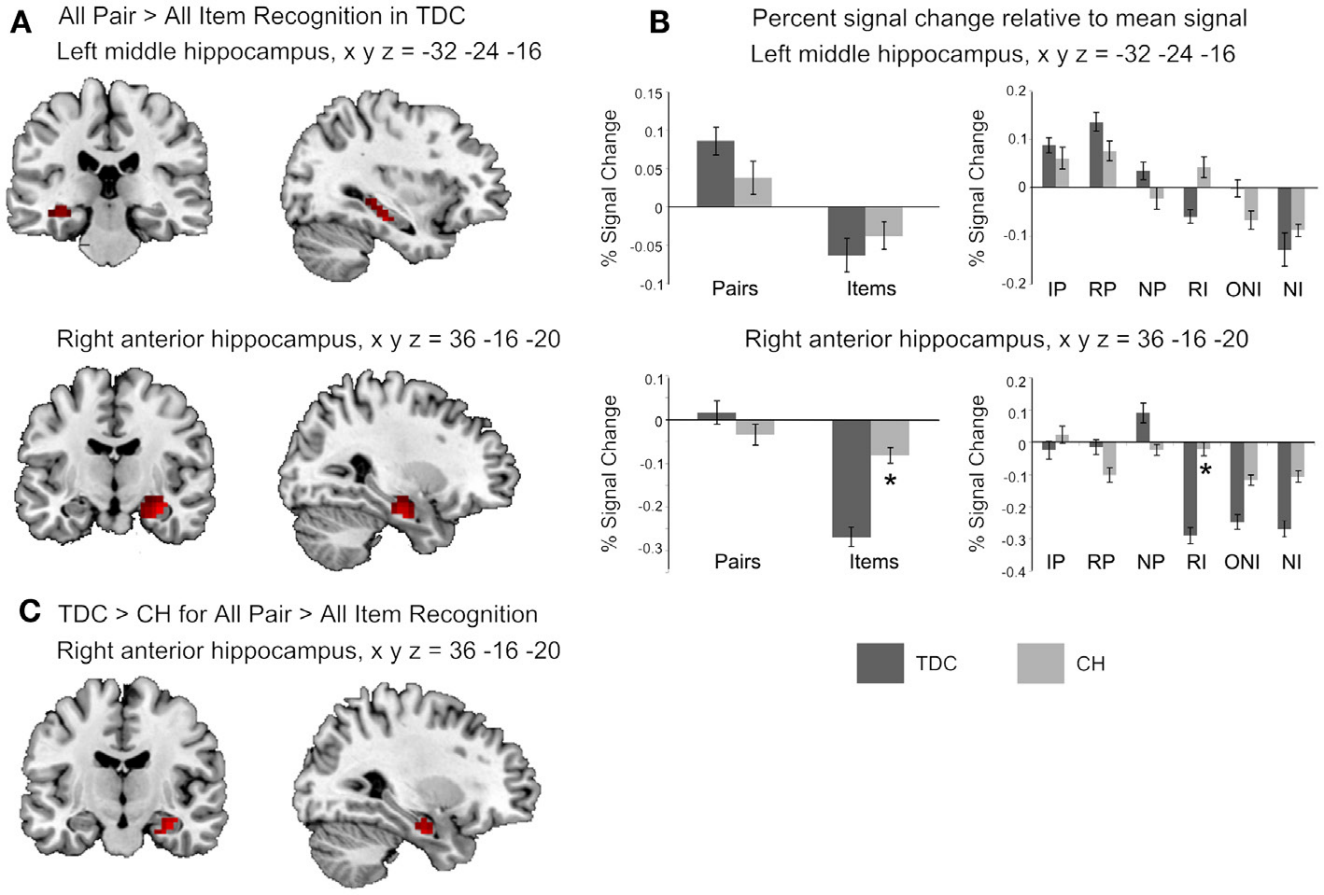
**Hippocampal regions identified in the fMRI analyses**. **(A)** The two clusters shown to be significantly more active in TDC for all Pair Recognition trials relative to all Item-Recognition trials (left middle hippocampus in top panel shown in both coronal and sagittal views, and right anterior hippocampus in the middle panel, again shown in both views). **(B)** Bar graphs illustrate mean percent signal change values for the two significant clusters and each set of bar graphs depicts these values for the peak coordinate of the cluster shown to its left. The left graphs show percent signal change for all Pair and all Item trials and the right graphs show values for each individual trial type (IP = intact pairs, RP = rearranged pairs, NP = new pairs, RI = rearranged items, ONI = one new item, NI = new items). **(C)** The right hippocampal region found to be significantly more active in TDC for Pairs versus Items than CH. Activation is shown at *p* < 0.05 uncorrected for visualization purposes, masked to include voxels only within the hippocampus, and overlaid on the ch2better.nii template in MRIcron. Peak coordinates (*x y z*) are given in Montreal Neurological Institute (MNI) space).

The fMRI contrast comparing all Pair-Recognition with all Item-Recognition trials showed, for TDC, significantly greater hippocampal activation in left-middle and right anterior hippocampal regions (see Figure 1; Table 3). In contrast, CH showed no clusters of increased hippocampal activation. Histograms showing percentage signal change for both tasks and individual trial types are presented in Figure 1. In the left middle hippocampus, both groups showed activation for previously seen words pairs and deactivation for items but the difference was more pronounced in TDC. In the right anterior hippocampus, TDC showed deactivation during Item trials and activation during novel Pair trials but not intact or rearranged Pair trials, whereas CH only showed deactivation for Item trials and to a significantly lesser degree than TDC.

**Table 3:** **Regions of the hippocampus more active during all Pair Recognition trials than all Item-recognition trials**.

		Cluster size	MNI coordinates		

Group	Hemisphere	*k*	*X*	*Y*	*Z*	*z-*score	*p*_uncorrected_	*p*_FWE_
TDC	R	23	36	−16	−20	4.30	<0.001	0.002
			32	−8	−28	3.46	<0.001	0.05
	L	9	−32	−24	−16	3.54	<0.001	0.04
			−32	−32	−4	2.69	0.004	0.35
CH	*ns*							
TDC > CH	R	6	36	−16	−20	2.88	0.002	0.237
CH > TDC	*ns*							

*Voxel-level *p*-values are provided, and cluster size (*k*) indicates the number of voxels comprising the cluster*.

*MNI, Montreal Neurological Institute; L/R, left/right; FWE, family wise error; X, left/right direction; Y, anterior/posterior direction; Z, superior/inferior direction*.

Between-group comparisons for the first contrast between Pair and Item trials indicated the difference was significantly larger in TDC than CH in the right anterior hippocampus (Table 3). Overall, it is clear that the profile of activation in the hippocampus is more selective to processing demands and memory conditions in TDC than in CH.

Multiple regression results indicated no early disease indices (age at treatment onset, TSH and T4 at diagnosis) significantly predicted hippocampal activation for the contrast between Pair and Item trials.

## Discussion

We investigated hippocampal function during verbal associative memory recognition in youth with CH. We hypothesized that CH and TDC groups would both show greater hippocampal activation when correctly recognizing word pairs versus individual words, as has been found for healthy adults using this paradigm (20). Based on our prior study with this group and visual associative memory (18), we expected that the increase for pairs versus items would be larger for CH than TDC and that early CH severity would predict both associative memory performance and atypical hippocampal functioning. Contrary to expectation, when TDC were recognizing word pairs versus individual items, they recruited bilateral hippocampus to a much greater degree than CH. Group effects were significant in the right anterior hippocampus. Markers of early disease in CH predicted overall memory accuracy but did not predict hippocampal engagement during pair versus item trials. Since these findings suggest youth with CH fail to recruit the hippocampus when remembering verbal associations to the same degree as TDC, this implies a lasting effect of early TH deficiency on later hippocampal functioning.

Percent signal change findings showing left and right hippocampal activation for each trial type give insight into specific cognitive processes differentiating the groups. Both groups engaged the left middle hippocampus when evaluating associations between previously seen but not novel words, while signal in this region was attenuated for individual items; this finding signifies a potentially distinct role of the left middle hippocampus in retrieving old associative details from memory. Since this region was active when pairs were both intact and rearranged, it appears to be involved in “flexibly” retrieving associations even when the stimuli are not presented exactly as studied (27). CH did not activate this region to the same extent as TDC, and this result suggests they may have reduced function in this region, possibly due to its smaller size (17).

Within the right anterior hippocampus, TDC exhibited strong signal reductions during individual item trials, whereas this deactivation was much less strong in CH. This discrepancy possibly signifies that CH were less able than TDC to inhibit hippocampal associative processing on trials requiring them to attend only to individual items. However, as we cannot be confident that deactivation reflects neuronal inhibition (28), this idea remains speculative.

Previously, we reported that during recognition of object pairs versus individual objects, youth with CH showed increased hippocampal activation relative to controls, particularly on the right side (18). In the present study, group differences were only found in the right hippocampus. This right-sided lateralization contrasts with other studies showing greater left than right hippocampus vulnerability, as we observed in our study showing CH had reduced left hippocampal volumes that also reflected their poorer verbal memory performance (17). In our visuospatial fMRI study, we found CH activated their left hippocampus more than peers when retrieving object-pair locations than individual objects (18). Since clinical findings (29) suggest CH have greater difficulty on verbal memory (associated with left hippocampus) than visual memory tasks (associated with right hippocampus) (30), the difference between our two fMRI studies may reflect the fact that CH participants were able to compensate for visual memory weaknesses by relying on both hippocampi in order to achieve equivalent performance to TDC, whereas on verbal memory tasks, which require the left hippocampus primarily, they may show a stronger impairment as this structure is reduced in size. This idea is corroborated by the fact that in the current study, the CH group had fewer “hits” or correct identifications of previously seen stimuli than TDC did. Even though the left hippocampus was not currently implicated in the between-group comparison, CH did show reduced fMRI signal in the left hippocampus during Pair trials relative to TDC.

We hypothesized that atypical hippocampal activation during verbal associative recall would reflect initial hypothyroidism severity. Our negative findings were unexpected since previous research has shown TH levels at diagnosis do predict later cognitive outcomes (31), particularly memory weaknesses (14, 16, 32). However, our behavioral results did show that the longer it took for a diagnosis to be made and treatment given (i.e., the longer the postnatal TH insufficiency), the poorer the memory performance, particularly on associative trials. The lack of association with fMRI data may reflect insufficient power since we observed hippocampal clusters that fell just below the minimum cluster extent threshold for significance in our regression analyses. Had we used a larger sample, we may have been able to detect these effects in fMRI data.

A possible mechanism for the effects of early postnatal hypothyroidism on later memory performance was recently proposed. Navarro et al. (33) showed that in rats with both developmental and early postnatal hypothyroidism, there were alterations within the basic hippocampal excitatory trisynaptic loop, with decreased VGluT1-ir button density in the dentate gyrus stratum distal-inner moleculare and in the CA1 hippocampal subfield stratum lacunosum-moleculare, with corresponding behavioral abnormalities. Such reduction in excitatory inputs to the hippocampus from the entorhinal cortex could interfere with the established role of the hippocampal subfields in encoding and retrieval of episodic memory sequences (34–36) and if a parallel process occurs in children with CH, it could underlie our current findings of persistent alterations in hippocampal function.

Our study investigated hippocampal group differences specifically, with slice coverage optimized for imaging the medial temporal lobe and not extending fully to prefrontal and parietal regions. However, it is also possible that there are differences in other brain areas, especially those with functional and anatomical connections to the hippocampus. For example, the dorsolateral prefrontal cortex is essential for formation of inter-item associations (37). Additional research is required to determine whether neural processing is altered more generally in CH. It is also unknown whether the observed hippocampal differences are stable or change with development in TDC and CH groups (38).

Study limitations include not obtaining TH levels at time of testing as this can affect cognitive functioning, particularly attention (39). Information on CH treatment adequacy was unavailable while TH values at birth were missing for some control subjects, although normal in the others. Maternal thyroid function data during pregnancy was also not collected as CH is not diagnosed until birth, although it would have been ideal to have this information as maternal hypothyroidism predicts later cognitive delays in offspring (40). Sample size was small for behavioral analyses, albeit acceptable for fMRI studies, signifying behavioral results must be interpreted cautiously. However, small effect sizes suggest the lack of significance in accuracy and RT was not a type-2 statistical error.

To conclude, we found that when adolescents with CH perform a verbal associative memory task, they display an atypical hippocampal response for paired words, characterized primarily by poorer discrimination in engagement during decisions about word pairs versus single items. This finding suggests that the neural mechanisms underlying associative binding are disrupted by early TH insufficiency. Overall, our results signify long-lasting effects of CH on hippocampal functioning.

## Author Contributions

SW, ES, MM, and JR conceived of and designed the study, and SW and ES acquired the data. VM, SW, and JR analyzed and interpreted the data as well as drafted the manuscript. All authors critically revised the manuscript and approved the final version for publication.

## Conflict of Interest Statement

The authors declare that the research was conducted in the absence of any commercial or financial relationships that could be construed as a potential conflict of interest.
